# Effect of Neem Leaf Extract and Neem Oil on *Penicillium* Growth, Sporulation, Morphology and Ochratoxin A Production

**DOI:** 10.3390/toxins1010003

**Published:** 2009-07-23

**Authors:** Simone A. G. Mossini, Carla C. Arrotéia, Carlos Kemmelmeier

**Affiliations:** Departamento de Bioquímica, Universidade Estadual de Maringá. Avenida Colombo, 5790, 87020-900, Maringá, PR, Brazil; Email: simonegmossini@yahoo.com.br (S.A.G.M.); ccarroteia@uem.br (C.C.A.)

**Keywords:** ochratoxin A, *Penicillium*, *Azadirachta indica*, biological control

## Abstract

*In vitro* trials were conducted to evaluate the effect of *Azadirachta* *indica* (neem) extracts on mycelial growth, sporulation, morphology and ochratoxin A production by *P. verrucosum* and *P. brevicompactum*. The effect of neem oil extract from seeds and leaf was evaluated at 0.125; 0.25 and 0.5% and 6.25 and 12.5 mg/mL, respectively, in Yeast Extract Sucrose (YES) medium. Ochratoxin A production was evaluated by a thin-layer chromatography technique. Oil extracts exhibited significant (p ≤ 0.05) reduction of growth and sporulation of the fungi. No inhibition of ochratoxin A production was observed. Given its accessibility and low cost, neem oil could be implemented as part of a sustainable integrated pest management strategy for plant disease, as it has been shown to be fungitoxic by inhibition of growth and sporulation.

## 1. Introduction

The presence and growth of fungi in food and feeds may cause spoilage and result in a reduction in quality and quantity. Fungi produce a variety of secondary metabolites as products of their metabolism; mycotoxins are metabolites that have deleterious effects on other organisms. Ochratoxin A (OTA), a potent mycotoxin produced by certain species of *Aspergillus* and *Penicillium*, originally associated with mouldy legumes, fruits, meat and cereal products, is at present receiving increasing attention for its nephrotoxic effects and its potential carcinogenic activity [1,2,3]. Cereal products are the major group of food commodities in which the above toxin is of high impact. Control techniques that are cheap, ecologically sound and environmentally safe to eliminate or reduce the incidence of economically important pathogens are of great importance, so the search for substances meeting these needs is an important research topic.

In recent years much attention has been given to the preservation of grains by natural products so that the growth and mycotoxin production may be effectively retarded. Non-chemical systems based on plant extracts for the treatment of microbial cultures have played an important role in the inhibition of pathogen growth and food quality improvement [4].

*Azadirachta indica* A. Juss (Meliaceae) (neem plant) extract is one of the most important plant products which inhibit mycotoxin production. It comprises several parts, such as fruit, seed, leaf and oil, with several active compounds [5,6,7]. The main chemical fractions of neem oil with antifungal activities are a mixture of triterpenoidal and tetranortriterpenoid compounds. Azadirachtin, 6-deacetyl-nimbin, azadiradione, nimbin, salannim and epoxyazadiradione were the major compounds obtained from chemical fractions of neem oil. When tested alone they did not have any appreciable activity, but when mixed they showed antifungal activity, indicating possible additive/synergistic effects [7]. The ability to cause retardation of fungus growth is the basic mode of action of a number of classical antifungal agents. However, neem leaf constituents are known to potentially inhibit aflatoxin production in *Aspergillus parasiticus* without affecting fungal growth [8,9]. According to Razzaghi-Abyaneh *et al.* [10], the main feature of neem extracts, particularly those derived from leaves, is that they do not retard fungal growth, but appear to interfere with aflatoxin production.

Data on fungal growth and mycotoxin production on the presence of neem extracts, apart from aflatoxins, are scanty, but “*in vitro*” studies with neem extracts showed inhibition of the polyketide mycotoxins patulin [11,12], citrinin [13], sterigmatocystin [14], but no inhibitory effect on penicillic acid [14] and fumonisin production [15].

The current study evaluates the effects of oil from neem seeds and of neem leaf extract on the growth, sporulation and ability to produce OTA by *Penicillium verrucosum* and *P. brevicompactum*.

## 2. Results and Discussion

**Figure 1:**
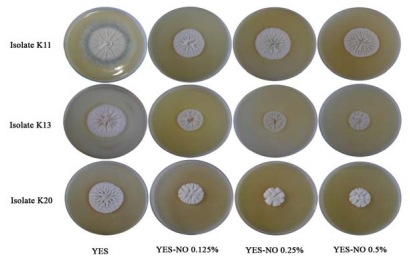
*P. verrucosum* (isolates K11, K13) and *P. brevicompactum* (isolate K20) on YES and on YES-Neem Oil (YES-NO) as described in ‘Materials and Methods’.

Neem seed oil (NO) and neem leaf extracts (NL) did not show the same effects on fungi. Direct contact of fungus with NO and NL on YES medium resulted in differences between the macroscopic features of the colonies but not in the microscopic ones. Colonies of fungi grown on YES-NO had practically the same size and appearance as those of controls (YES): white color, radially sulcate, moderately deep and produced exudates (Figure 1).

**Figure 2:**
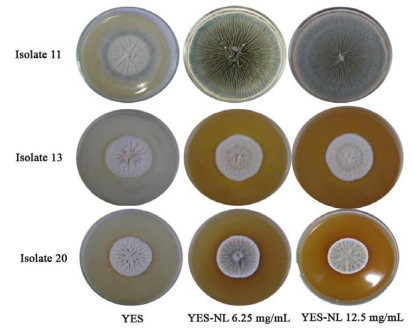
*P. verrucosum* (isolates K11, K13) and *P. brevicompactum* (isolate K20) on YES and on YES-Neem Leaf Extracts (YES-NL) as described in “Materials and Methods”.

Fungi colonies grown on YES-NL were not only bigger in size than control (YES), but their appearance differed too: green color, radially sulcate, lightly deep and produced more exudates (Figure 2).

Microscopic observations revealed same size of conidia and conidiophores; there was no alteration in asexual reproduction development, typical branching and appearance of conidiophores (results not shown). Comparisons were also made with morphologic features of *P. verrucosum* and *P. brevicompactum* described in literature [16].

There was no difference in spore area between K11 control and K11 treatments (p ≥ 0.05). For isolate K13 and K20, all groups, when compared, were significantly different (p < 0.001). However, there was no similar alteration of the spore area in the isolates. Whereas in K13 the NO extracts increased their spore area, NL extracts decreased theirs; in K20 the NO and NL extracts decreased their spore area (Table 1).

**Table 1:** Effects of Neem extracts on *Penicillium* species (isolates K11, K13 and K20).

FUNGUS		NEEM EXTRACTS					
		Neem Oil (NO)				Neem Leaf Extract (NL)	
		Control^a^	NO 0.125%^a^	NO 0.25%^a^	NO 0.5%^a^	NL 6.25 mg/mL^a^	NL 12.5 mg/mL^a^
K11	Dw (g)^a^	0.094 ± 0.001	0.079 ± 0.001	0.082 ± 0.002	0.073 ± 0.002	0.143 ± 0.001	0.119 ± 0.001
	Dw -R (%)	-	15.95%^*^	12.76%^*^	22.34%^*^	0%	0%
	D (mm)^a^	7.08 ± 0.52	3.66 ± 0.08	3.56 ± 0.20	3.43 ± 0.16	8.75 ± 0.24	8.16 ± 0.54
	D - R (%)	-	48.30%^**^	49.71%^**^	51.55%^**^	0%	0%
	S (cm^2^)^a^ (x10^7^)	1.3 ± 0.15	0.7 ± 0.04	2.1 ± 0.11	2.9 ± 0.2	7,3 ± 1.05	19.7 ± 1.5
	S - R (%)	-	47.24%^**^	0%	0%	0%	0%
	Sa (μm^2^)^b^	10.48 ± 0.20	NA	10.32 ± 0.21	NA	NA	10.58 ± 0.18
	Sa-R (%)	-	-	4.79%	-	-	2.39%
	OTA^a^	+	+	+	+	+	+
K13	Dw (g)^a^	0.098 ± 0.001	0.077 ± 0.002	0.069 ± 0.002	0.083 ± 0.001	0.101 ± 0.002	0.086 ± 0.002
	Dw -R (%)	-	21.42%^*^	29.59%^*^	15.30%^*^	0%	12.24%
	D (mm)^a^	4.06 ± 0.08	2.7 ± 0.16	2.48 ± 0.04	2.5 ± 0.08	3.71 ± 0.14	3.62 ± 0.09
	D - R (%)	-	33.5%^**^	39%^**^	38.4%^**^	8.62%	10.8%^*^
	S (cm^2^)^a^ (x10^7^)	2.8 ± 0.14	0.5 ± 0.04	0.7 ± 0.04	1.4 ± 0.08	11 ± 0.17	13 ± 0.28
	S - R (%)	-	82.6%^**^	75.6%^**^	51.2%^**^	0%	0%
	Sa (μm^2^)^b^	4.73 ± 0.23	NA	7.23 ± 0.19	NA	NA	3.11 ± 0.11
	Sa-R (%)	-	-	0%	-	-	34.29%^**^
	OTA^a^	+	+	+	+	+	+
K20	Dw (g)^a^	0.13 ± 0.001	0.09 ± 0.001	0.089 ± 0.001	0.068 ± 0.001	0.111 ± 0.00	0.109 ± 0.00
	Dw -R (%)	-	30.76%^*^	31.53%^*^	47.69%^*^	15.38%^*^	16.15%^*^
	D (mm)^a^	3.68 ± 0.07	2.56 ± 0.02	2.35 ± 0.02	2.25 ± 0.05	3.4 ± 0.18	3.41 ± 0.07
	D - R (%)	-	30.43%^**^	36.14%^**^	38.85%^**^	7.60%	7.33%
	S (cm^2^)^a^ (x10^7^)	4.0 ± 0.31	0.8 ± 0.12	0.7 ± 0.09	4.0 ± 0.20	33 ± 1.58	43 ± 2.87
	S - R (%)	-	80.09%^**^	82.58%^**^	0.24%	0%	0%
	Sa (μm^2^)^b^	11.5 ± 0.18	NA	6.66 ± 0.20	NA	NA	9.14 ± 0.13
	Sa-R (%)	-	-	42.03%^**^	-	-	20.45%^**^
	OTA^a^	+	+	+	+	+	+

Experimental details are as described in “Materials and Methods”. Dw: dry weight; Dw-R (%): dry weight reduction %; D: diameter; D-R (%): diameter reduction %; S: spores number; S-R (%): spores number reduction %; Sa: spores area; Sa-R (%): spores area reduction %; OTA: ochratoxin A (+) detected by TLC; NA: not analysed; R (%): were determined by: (mean control - mean treatment)/(mean control) × 100%; ^a ^Values, average ± standard error (n = 6); ^b^ Values, average ± standard error (n = 200); ^* ^Significant at *p* < 0.05; ^** ^Significant at *p* < 0.001.

**Figure 3:**
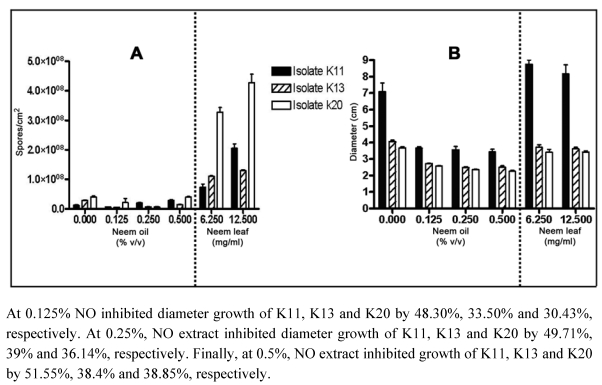
Effect of neem oil and neem leaf extracts on sporulation (A) and on colony diameter (B) of *Penicillium* (isolates K11, K13 and K20) on YES medium, determined as described in ‘Materials and Methods’. Bars standard deviation for experiments carried out in six replicates.

The ability of neem extracts to inhibit growth and sporulation varied widely. Results (Table 1 and Figure 3 A and Figure 3 B) shows that NO extract was effective for reducing fungal growth at all levels (p ≤ 0.05), however, NL extract was not only ineffective, but showed a stimulatory effect on isolate K11.

Inhibition of mycelial growth was generally associated with the inhibition of sporulation. However, neem extracts enhanced or increased sporulation, depending on the extract’s concentration, without showing the same associated effect on mycelial growth. NO extract significantly (p ≤ 0.05) inhibited the sporulation of all fungi at 0.125%, by 47.24%, 82.6% and 80.1% (K11, K13 and K20, respectively). NO (0.25%) inhibited (p ≤ 0.05) the isolates K13 (75.6%) and K20 (82.58%)and had a stimulatory effect on sporulation of isolate K11. NO (0.5%) significantly (p ≤ 0.05) inhibited the fungus K13 (51.2%) and stimulated K11. NL extract did not inhibit the sporulation of the fungus, but showed a stimulatory effect on all isolates. The effect of extracts on dry weight was also variable: NO decreased in all isolates; NL increased weight in K11 weight and decreased in K13 and K20 (Table 1).

The extracts’ different effects on radial growth, sporulation and mycotoxin production may be either due to the solubility of the active compounds or inherent to fungal metabolism. NO extracts showed higher inhibitory effects than NL, probably owing to the presence of azadirachtin at its highest concentration in the mature seeds [15,17].

**Figure 4:**
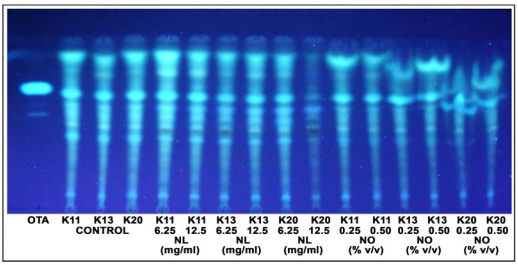
Thin Layer Chromatography of OTA production by *Penicilium* isolates (K11, K13 and K20) without neem extracts (Control) and with neem leaf (NL) and neem Oil (NO) extracts. Ultraviolet light visualization.

Toxin production generally decreases as mycelium formation decreases, but research has shown that antifungal potentiality against growth may not coincide with the inhibitory potential of toxin production. [5,9,11,18,19,20]. Current analyses revealed inhibitory potential of NO extracts on sporulation and mycelium growth of fungi, but not on OTA production (Figure 4).

Inhibition of this toxin production does not appear to be simply a function of mycelium weight or sporulation reduction. Although sporulation is typically accompanied by mycotoxin production, this is not always true, because mycotoxins may be produced at high levels under growth conditions where sporulation is inhibited [21].

Data on fungi growth and mycotoxins production on the presence of neem extracts, apart from aflatoxins, are scanty, but neem aqueous and oily extracts, in a similar range of concentrations as used in the present study, showed inhibitory effects when tested against some polyketide mycotoxins: patulin [11,12], citrinin [13], sterigmatocystin [14] production and also to aflatoxin [8,9,10,20], but were ineffective to penicillic acid [14], and now to ochratoxin A.

Neem extracts have fungitoxic activity, although their mode of action is not understood very well. It is quite possible that the different chemicals or different ratios of chemicals found in the neem trees have varied effects on fungi. There is also evidence from these studies and many others that fungal species react differently to compounds from the neem tree. The current research has shown that neem oil could be implemented as part of a sustainable integrated pest management strategy for plant disease, once neem oil has been shown to be fungitoxic to growth and sporulation.

## 3. Material and Methods

### 3.1. Mycotoxigenic Fungi

Ochratoxin A (OTA) producing isolates of *Penicillium verrucosum* (K11 and K13) and of *Penicillium brevicompactum* (K20), maintained in silica storage [22] at the culture collection of the Laboratory of Chemistry and Physiology of Microorganisms (Department of Biochemistry. State University of Maringá, Pr.), were used as test organisms.

### 3.2. Preparation of Neem Extracts

Oil from neem seeds (NO) was obtained from DalNeem Co., Brazil, and leaves of *Azadirachta indica* A. Juss (Meliaceae) were hand-collected from healthy mature trees at the Agricultural Institute of the State of Paraná (IAPAR) in Londrina, PR. Brazil. A 10% water extract from dried neem leaves (NL) was prepared by maceration [11].

### 3.3. Culture Conditions for Production of Ochratoxin A (OTA)

Yeast Extract Sucrose (YES) [23], recommended for extracellular mycotoxins [24], was the liquid medium used for OTA production. To examine the toxin production in pure culture, each of the *Penicillium* strains was first cultivated on Czapek yeast agar (CYA) [23] at 25°C for 7 days and then inoculated on YES. The YES cultures were incubated at 25°C for 7 days. Inocula containing 10^5 ^spores of each strain of *P. verrucosum* and *P. brevicompactum* were added to 3 mL of YES medium in 25 mL Pyrex^®^ flasks (20 × 100 mm) and incubated at 26 ± 0.5°C, for 7 days, without shaking. Treatments in four replicates consisted of 0.125, 0.25, 0.5% (v/v) of neem oil and 10% freeze-dried aqueous neem leaf extract at 6.25, 12.5 mg/mL, added to YES medium. Mould growth was reported visually throughout incubation and mycelial growth was measured daily until the end of the incubation period. Dry weights were obtained after extraction; mycelia were separated from the broth culture and transferred to preweighed filter paper, dried at 60°C until constant weight, to determine the amount of growth. OTA was extracted and purified from the broth by procedures according to Scott *et al*. [25]. Chloroform extracts were combined and dried by filtering through anhydrous sodium sulfate to remove water. Chloroform was evaporated to dryness and residue dissolved in chloroform.

### 3.4. Mycelial and Sporulation Inhibition Test

The effects of NO and NL on radial growth, colony characteristics and sporulation of *P. verrucosum* and *P. brevicompactum* were determined by growing on YES agar in the absence (control) and presence (treatments) of the neem compounds. These media were needle inoculated at the center of an inverted Petri plate. Fungi were previously cultured in YES agar in Petri dishes, for producing isolated colonies. Cultures were subsequently incubated at 25°C, for 7 days. Treatments consisting of NO (0.125, 0.25 and 0.5% v/v) and NL (6.25 and 12.5 mg/mL) were added to the solid media. Radial growth was measured daily until incubation time, compared and mean values taken, as described by Amandioha [26]. Macroscopic and microscopic morphological features (0.1% Lactofuchsin staining [27]) were taken and analyzed. Sporulation was measured according to Gusman-de-Pena and Herrera [28]. Briefly, mycelium grown in solid media was removed, transferred to a flask containing a sterile 0.1% Tween 80 solution (10 mL) and stirred with a vortex mixer for two minutes to free the spores. After mycelium sedimentation, the supernatant containing the spores was recovered and counted using a Neubauer counting chamber. Sporulation data were recorded by spores/cm^2^ of colony. Percentage inhibition of mycelial growth and sporulation by leaf extracts and oil were calculated as by Amandioha [26]. Spore diameter was analyzed for morphometric analysis with a biological optical microscope, 40 × lens, equipped with an IPPWIN-DCAM image-taking kit. An area (µm^2^) of 200 spores of each isolate (control and treatments) was measured by Image-Pro-Plus 3.0.1 software of image analysis. Values were analyzed by Turkey’s test (PRISMA software) and significance level was set at p < 0.05. Data were reported as mean (M) ± standard error (SE) for the indicated number of observations (n). Two experiments were performed with six replicates per treatment and with one control per experiment.

### 3.5. Ochratoxin A (OTA) Detection

Ochratoxin A (Sigma Chemical Co., St. Louis, MO, 98% purity) standard solution was prepared in UV spectroscopic grade ethanol (1 mg/mL) and stored at 4^o^C. OTA standard and residues of the extracts dissolved in chloroform were spotted (10 μl) on TLC plates (20 × 20 cm silica gel 60 - G on aluminum sheets, Merck) and developed using the solvent system: toluene: ethyl acetate: formic acid (5:4:1; v/v), as reported by Scott [29] followed by exposure of plate to ammonia fumes [30]. The ochratoxin-ammonia derivative appeared as a light-blue fluorescing spot under ultraviolet light. Confirmatory test for OTA was done by over-spotting with authentic standard and by conversion into a new fluorescent compound as described by Golínski [31]. The detection limit of OTA by TLC is about 8 µg/kg and 10 ppb as described by Gimeno [32] and DeVries *et al*. [33] respectively. All solvents used were analytical grade.

### 3.6. Statistical Analysis

All statistical analyses were performed by Turkey’s honest significant difference multiple comparison tests to segregate treatments, which were significantly different (PRISMA software). Analysis of variance (ANOVA) tests were performed for significant (p ≤ 0.05) differences between experiments.

## 4. Conclusions

In the current study the effects of neem extracts on growth, sporulation, morphology and OTA production by *Penicillium verrucosum* and *P. brevicompactum* were investigated. Thin layer chromatography failed to show any inhibition of OTA production; however, neem extracts affected the growth rate and sporulation of isolates. Assays show that neem extracts have fungitoxic activity, even though their mode of action is still not fully understood. It is also quite possible that different chemicals or different ratios of chemicals found in neem trees have varied effects on fungi. Evidence from the current and other studies shows that fungal species react differently to compounds from the neem tree. Additional research is needed to determine the potential usefulness of neem products in fungal control programs.
